# Development and Validity of the Fear of Water Assessment Questionnaire

**DOI:** 10.3389/fpsyg.2020.00969

**Published:** 2020-05-29

**Authors:** Fatmir Misimi, Tanja Kajtna, Samir Misimi, Jernej Kapus

**Affiliations:** Faculty of Sports, University of Ljubljana, Ljubljana, Slovenia

**Keywords:** swimming assessment, coaching, drowning, testing, swimming

## Abstract

Fear of water is the strongest predictor for no or low swimming competencies. Some individuals will never learn to swim due to their complete avoidance of water, whereas others might have difficulty with learning due to the fact that they cannot sufficiently relax their body to facilitate floating or swimming. Therefore, it is important to identify these people and to establish effective teaching strategies that can best help this specific population. Recognizing this, there is a clear need for an assessment tool which can help swim teachers and coaches identify people with a fear of water. The study aimed to first develop and then validate a fear of water assessment questionnaire (FWAQ). 2074 male and female people participated in the creation of a 40-item questionnaire. The exploratory factor showed that a 3 factor solution including 20 items was most sensible – such a solution accounted for 31.69% of explained variance and the Cronbach’s alpha α was 0.831, which makes for a reliable enough solution. A subsequent discriminant function analysis correctly classified 98.2% of participants. We concluded that the findings from this study support that the FWAQ is a valid scale that effectively identify people with fear of water.

## Introduction

Well-developed swimming skills are not only essential for drowning prevention, but also contribute to the development and maintenance of overall fitness (Brenner et al., 2009; Beggs et al., 2013). Learning how to swim is not only a physical, but also a cultural achievement (Majumder and Choudhury, 2014). Therefore, instructional swimming programs for both beginners and advanced swimmers form part of physical education curriculums at different levels of education in many European countries (Jurgec et al., 2016). Gilchrist et al. (2000) found that, despite many efforts to improve swimming knowledge, many people still don’t know how to swim. We still have little data about the swimming abilities of children and adolescents, and the available data are inadequate and unable to be compared between different countries due to varying methodological approaches. By using an interview and examination survey, data illustrated that 14.5% of 5- to 17-year-olds in Germany were unable to swim (Kuntz et al., 2016). Moreover, in the United Kingdom, approximately half of children aged 7–11 years of age were found to be incapable of swimming 25 m (Amateur Swimming Association, 2013). Nearly 64% of African American children, 45% of Hispanic children, and 40% of Caucasian children have little or no swimming ability (The United States Association Swimming Foundation, 2017). Data from the Ministry of Education, Science and Sport of the Republic of Slovenia illustrate that 7% of people 12 years of age cannot swim 50 m, thus they are classified as non-swimmers (Grujić, 2018).

### Theoretical Background

There are a variety of reasons why many children and adolescents cannot swim (Pharr et al., 2018). In children and adults who avoid swimming lessons there exists barriers which makes them avoid swimming. Such barriers may include accessibility to the pool, cultural issues of not wanting to learn to swim, racial and ethnic factors such as hair care, discomfort of being seen in swimsuits, parents whose fear of water could discourage their children from learning to swim, injuries that happened to family and friends, drowning, illness, and/or negative experiences (Lachocki, 2012). However, fear of drowning is a very common factor (Berukoff and Hill, 2010; Pharr et al., 2018). Indeed, it is the strongest predictor of no or low swimming ability (i.e., even stronger than family finances or access to swimming facilities) (Ziara, 2005; Irwin et al., 2010). The fear of drowning could originate within the common overall fear of water (Whiting and Stembridge, 1965; Shank, 1987). Fear of water or aquaphobia is considered to be a “specific phobia,” which means “a marked and persistent fear that is cued by circumscribed clearly discernible objects or situations” (American Psychiatric Association [APA], 2000, pp. 219), The prevalence rate of aquaphobia in the general population is between 2 and 3% (Stinson et al., 2007), and it is more common among children than adults (Menzies and Harris, 1997). Specific phobias, such as a fear of water, usually originate from childhood and are frequently intensified through adulthood (Becker et al., 2007). The origins of a fear of water during childhood has been examined. The most common belief is that it is usually linked to a previous bad experience (Whiting and Stembridge, 1965; Shank, 1987). These could be bad swimming lessons, an accidental fall into deep water, near drowning experiences, etc (Shank, 1987). In contrast, it has also been suggested that the origins of the fear of water can best be explained by non-associative processes (Menzies and Clarke, 1993; Graham and Gaffan, 1997). This means that it mainly reflects a biological fear that manifests often without aversive experiences (Poulton et al., 1998). A broad range of situations may elicit the fear of water, such us being or swimming in water that is dark or opaque (i.e., without clear vision of what is in the water), submerging one’s head below the water, being near fountains, and traveling on a boat (Milosevic and McCabe, 2015). Rarely, even bathing may provoke a fear response to water. Considering this, the fear of water can disrupt a number of activities which are carried out in water or near it (Milosevic and McCabe, 2015). Some individuals never learn to swim due to their complete avoidance of water, whereas others might have difficulty with learning due to the fact that they cannot sufficiently relax their body to facilitate floating or swimming (Milosevic and McCabe, 2015). Therefore, it is reasonable to conclude that the fear of water is a considerable factor, which may put children or adolescents at a high risk of drowning (Irwin et al., 2015). These individuals are the most likely to panic if they find themselves in a dangerous situation, which is a key determinant of fatal or nonfatal drowning events (Grenfell, 2004).

372000 people die from drowning every year, which makes drowning a serious public health threat and one of the ten leading causes of death in children under 5 years of age and young males between 15 and 19 years (World Health Organization [WHO], 2014). Learning to swim and education on water safety are two of the most important strategies for preventing drowning (Leavy et al., 2019), some authors especially emphasize the protective value of swimming ability against drowning (Brenner et al., 2009). Data from the United States show that 74% of drowning victims didn’t know how to swim (Cody et al., 2004), similarly over one third of drowning victims between 5 and 14 years of age in Canada had no or poor swimming abilities (Canadian Red Cross, 2003). There are also interesting findings regarding good swimming abilities - better swimming ability has been found to be related to more water-related risk behaviors (Ma et al., 2010), which was translated into higher drowning risk by overconfidence in the water (Smith, 1995).

Collectively, it is important that people with a fear of water learn how to swim. Moreover, it is important to identify them and to establish effective teaching strategies that can best help this specific population (Stillwell, 2011). Recognizing this, there is a clear need for an assessment tool which will help swimming teachers and coaches identify those with a fear of water. Therefore, the aim of the current study was to develop and to validate the fear of water assessment questionnaire (FWAQ).

Three studies were conducted to test our adapted FWAQ. In Study 1, we composed the items and looked for the factor structure of the FWAQ, allowing important items to be retained and subsequently interpreted. In Study 2, we assessed the reliability of the FWAQ. Finally, in Study 3, we examined the discriminant function of the FWQA. All studies were approved by an ethical committee and were conducted in accordance with the Helsinki Declaration.

## Study 1 Item Generation and Exploratory Factor Analysis

### Methods

#### Item Generation

To develop the items for FWAQ, an interdisciplinary team of researchers from the sport sciences was used, which included three experts from different fields: A sport psychologist, a swim pedagogue, and a swimming coach experienced in teaching swimming were assembled to accomplish this project. The following is a survey designed with a list of 40 items (Table 1).

**TABLE 1:** Item generation.

**Item Number**	**Statements**	**Completely disagree**	**Disagree**	**I am not sure**	**Agree**	**Completely agree**
1	I think I could get lost in the sea during swimming	1	2	3	4	5
2	When I see waves, I get scared	1	2	3	4	5
3	When I see open water on the sea, I feel fear	1	2	3	4	5
4	I could swallow water when waves hit me	1	2	3	4	5
5	I am afraid of fish in the sea	1	2	3	4	5
6	My eyes could fill up with water and cannot see	1	2	3	4	5
7	When I submerge with my face, I am worried I could swallow water	1	2	3	4	5
8	I could not swim in the river because of flowing water	1	2	3	4	5
9	When I am in a pool, I am afraid when I am not in contact with floor	1	2	3	4	5
10	I am afraid when the water is deep	1	2	3	4	5
11	I am able to cope with an unexpected and involuntary submersion in water	1	2	3	4	5
12	When I am in the pool, I am afraid to open my eyes in water	1	2	3	4	5
13	When I am in the pool, I am afraid to put my face in the water	1	2	3	4	5
14	When I start to swim in the pool, I am afraid to see how far the finish edge is	1	2	3	4	5
15	When I am in the pool, I am afraid to swim when I see a lot of people	1	2	3	4	5
16	When I am in the pool, I am afraid to swim when I am alone	1	2	3	4	5
17	I cannot swim without goggles	1	2	3	4	5
18	I cannot swim with goggles	1	2	3	4	5
19	I feel safe when I use swim board	1	2	3	4	5
20	I do not feel good when I use swim board	1	2	3	4	5
21	I did not learn how to swim because my home is far away from: swimming pool, lake, river or sea	1	2	3	4	5
22	I need to learn how to swim because of water safety	1	2	3	4	5
23	My parents told me that it is dangerous to swim in deep water	1	2	3	4	5
24	I am afraid when I lift my legs and float on the surface	1	2	3	4	5
25	I do not like to jump into the water	1	2	3	4	5
26	I need stairs or shallow water to enter the water	1	2	3	4	5
27	I should clean my eyes when my face was in the water	1	2	3	4	5
28	I could not make bubbles	1	2	3	4	5
29	I swim with face above the water	1	2	3	4	5
30	I could not swim front crawl because of breathing	1	2	3	4	5
31	Swimming on back is easy because of breathing	1	2	3	4	5
32	When my legs sink, I am afraid	1	2	3	4	5
33	I am able to push from the wall and glide on the surface	1	2	3	4	5
34	I am able to jump legs first into the water from starting block	1	2	3	4	5
35	I am able to jump head first into the water from starting block	1	2	3	4	5
36	I am able to pick up things from the bottom of the shallow pool	1	2	3	4	5
37	I am afraid to touch the bottom of the 2 m deep pool and not being able to breathe	1	2	3	4	
38	I am able to pick up things from the bottom of the 2-meter-deep pool	1	2	3	4	5
39	I am able to dive and to swim under the surface of 5 m	1	2	3	4	5
40	I am uncomfortable when someone is splashing me	1	2	3	4	5

#### Participants

Two thousand seventy four participants [1002 males and 1072 females, ages ranging from 13 to 76 years (average age was 24.5 ± 11.7 years)] participated in the study. They were extensively informed regarding the aims of the study before providing their written consent. Participants were from the cities Mitrovica, Peja, Gjakova, Prizren, Ferizaj, Gjilan, Pristina, and Podujevo in the Republic of Kosovo. These cities were selected due to their diverse populations.

#### Procedure

The interviewers stood in the main street of each city and selected every third person who came by. They tried to sample similar numbers of men and women. We asked the participants to fill out the FWAQ. They rated each item according to their degree of agreement or disagreement by using a five-point Likert scale (1 = strongly disagree, 2 = disagree, 3 = not sure, 4 = agree, 5 = strongly agree).

#### Data Analysis

We used exploratory factor analysis (EFA) with principal components analysis (PCA) with a Varimax rotation. Data was analyzed with the SPSS 20.0 package (SPSS Inc., Chicago, United States).

### Results

Sample size turned out to be large enough for a factor analysis (KMO = 0.912), this decision was supported by Bartlett’s test of sphericity’s significance (χ^2^ = 12304.337; df = 780; *p* = 0.000).

Item communalities ranged from 0.308 to 0.651 (*M* = 0.496), we decided on a 3-factor solution based on the eigenvalues using the Kaiser criterion, even though the percentage of explained variance is rather low – this structure accounted for 31.69% of the total variance. Eigenvalues ranged from 18.9 to 4.73.

#### Relationships Between Factors

We examined both the pattern matrix and the structure matrix – Table 2 shows factor loadings on each item, whilst correlations between factors are shown in the structure matrix.

**TABLE 2:** Factor loading.

**Item number**	**Factor**								
	**1**	**2**	**3**	**4**	**5**	**6**	**7**	**8**	**9**
16	0.666								
15	0.652								
13	0.645								
17	0.639								
14	0.630								
12	0.530								
24	0.404								
26	0.399								
21	0.395								
2		0.725							
3		0.707							
1		0.629							
9		0.489							
10		0.478							
8		0.392							
32		0.391							
34			0.764						
35			0.736						
36			0.637						
33			0.628						
31				0.623					
22				0.598					
27				0.568					
23				0.554					
29				0.444					
6					0.683				
7					0.631				
4					0.512				
5					0.507				
28						0.716			
30						0.497			
25						0.450			
39							0.642		
38							0.585		
11							0.549		
37							−0.316		
40								0.670	
19								0.461	
18									0.732
20									0.719
Eigenvalues	7.56	3.22	1.89	1.45	1.29	1.16	1.13	1.07	1.02

#### Interpreting and Naming the Factors

Following the recommendations of Tabachnick and Fidell (2013), we kept the items with a factor loading of more than 0.32 and the meaning of each factor was based mostly on the strongest loading items. Thus we kept 20 items and, according to the recommendations of Henson and Roberts (2006), we conducted a second EFA on the remaining 20 items. The 3-factor structure was confirmed, accounting for 40% of the total explained variance.

## Study 2 Reliability of the FWAQ

### Methods

#### Participants

We used the same participants and data as in Study 1 in order to test the internal consistency of FWAQ.

#### Data Analysis

We calculated Cronbach’s alpha coefficients, data was analyzed with the SPSS 20.0 package (SPSS Inc., Chicago, United States).

### Results

A Cronbach’s alpha of 0.831 was determined, which makes for a very good internal consistency of FWAQ. Internal consistency for Factor 1 was 0.805, Factor 2 was 0.798, and 0.71 for Factor 3.

## Study 3 Examining the Discriminant Function of the FWAQ

We wanted to test if the FWQA could effectively discriminate between “Fear of water” and “No fear of water” participants.

### Methods

#### Participants

One hundred and ten participants (53 males, 57 females), ages ranging from 10 to 12 years of age (average age was 10.5 ± 0.5 years), participated in the study.

#### Procedure

Ethical approval was granted from the authors and the study was carried out in accordance with the Declaration of Helsinki for ethical principles. Participants and their parents were extensively informed regarding the aims of the study before written consent of the parents was provided. We asked the participants to fill out the FWAQ. They rated each item according to their degree of agreement or disagreement by using five-point Likert scale (1 = strongly disagree, 2 = disagree, 3 = not sure, 4 = agree, 5 = strongly agree).

When the data was collected, a physical education teacher with experience of teaching swimming was asked to be an evaluator. They asked participants to rate their responses on a five-point Likert scale based on imagining themselves in water-area scenarios. Those ranked “Completely disagree” or “Completely agree” on the subjective rating scale were classified as “No fear of water” (*n* = 58), those who scored 4 or 5 on the scale were classified as “With fear of water” (*n* = 52).

#### Data Analysis

To test for differences between groups we calculated MANOVA and used the level of significance at *p* < 0.05 (Field, 2018) and then a discriminant function analysis to check for prediction of group membership. We used the SPSS 20.0 package (SPSS Inc., Chicago, United States).

### Results

A Mahalanobis distance of 43.10 was calculated, which is below the critical value of 45.31, suggesting multivariate normality (Tabachnick and Fidell, 2013). Correlations between the variables ranged from 0.189 to 0.553 and we found a difference in FWAQ scores between the groups with fear of water and no fear of water, [*F*(20.89) = 31.21, *p* < 0.005, Wilks Lambda = 0.125, partial eta squared = 0.875]. Descriptive statistics of final FWAQ scores of fear of water and no fear of water groups, effect sizes, and significance levels of differences between groups are presented in Table 3.

**TABLE 3:** Discriminant function.

**Item number**	**Fear of water group mean (±SD)**	**No fear of water group mean (±SD)**	**Effect size**	**Significance**
1	4.02 (1.244)	1.88 (1.272)	0.423	*p* < 0.001
2	3.19 (1.534)	1.24 (0.683)	0.416	*p* < 0.001
3	3.67 (1.491)	1.40 (1.075)	0.442	*p* < 0.001
4	3.75 (1.519)	2.21 (1.587)	0.200	*p* < 0.001
5	3.79 (1.576)	1.45 (0.921)	0.462	*p* < 0.001
6	4.17 (1.294)	1.83 (1.391)	0.435	*p* < 0.001
7	3.71 (1.391)	1.31 (0.754)	0.547	*p* < 0.001
8	3.92 (1.453)	1.40 (0.897)	0.533	*p* < 0.001
9	4.02 (1.421)	1.57 (1.299)	0.452	*p* < 0.001
10	3.58 (1.526)	1.22 (0.702)	0.508	*p* < 0.001
11	3.98 (1.526)	1.53 (1.063)	0.486	*p* < 0.001
12	3.96 (1.267)	2.03 (1.226)	0.344	*p* < 0.001
13	4.52 (0.918)	1.57 (1.094)	0.682	*p* < 0.001
14	4.54 (0.939)	1.81 (1.249)	0.604	*p* < 0.001
15	3.63 (1.495)	1.86 (1.35)	0.282	*p* < 0.001
16	3.62 (1.598)	1.69 (1.217)	0.321	*p* < 0.001
17	1.92 (1.234)	4.26 (1.371)	0.447	*p* < 0.001
18	1.58 (1.073)	4.26 (1.208)	0.582	*p* < 0.001
19	1.63 (0.991)	4.45 (1.012)	0.667	*p* < 0.001
20	1.54 (1.075)	4.07 (1.400)	0.507	*p* < 0.001

*Responses were on a 5-poit Likert scale from 1 (“strongly disagree”) to 5 (”strongly agree”).*

An initial examination of the variables means show that those in the groups with a fear of water scored better (i.e., higher on the first factors, and second factor while lower on the third factor), suggesting that those people rated as more likely to have a fear of water. Twenty of the variables showed statistically significant level of 0.001. Bonferroni adjustment testing resulted in similar significance, alpha 0.001.

**FIGURE 1:**
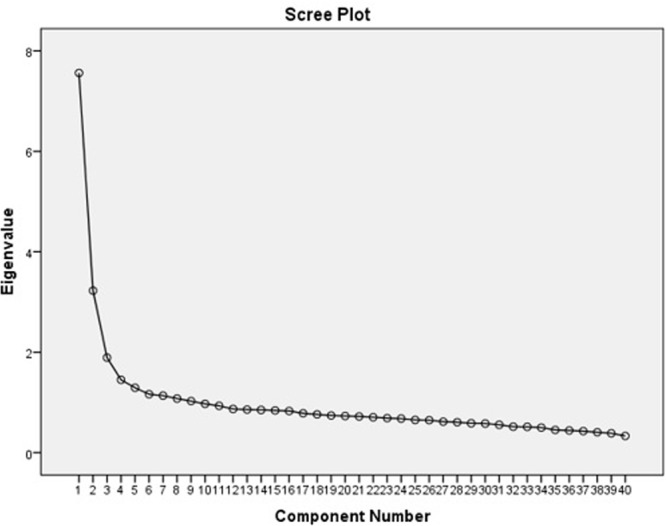
Scree plot of the factor analysis.

We found a significant discriminant function of the FWAQ (Wilks Lambda = 0.125, χ^2^ = 203.975, *p* < 0.001), with a canonical correlation of 0.936. The FWAQ was able to correctly predict 98.18% (51 out of 52) of the “with fear of water” group members and 98.3% (57 out of 58) of the “no fear of water” group members; in total 98.2% of the 108 participants could be correctly classified. The standardized canonical discriminant function coefficients show that factors one and two (active coping) and factor three (adverse response to fear of water) are important for group differentiation as can be seen from Figure 1.

## Discussion

The aim of this study was to develop and to validate the FWAQ. The final FWAQ consisted of 20 items (Appendix Table A1) and includes three factors, which account for 31.69% of the total variance. Discriminant function analysis shows that the FWAQ can correctly classify participants as having or not having fear of water, which suggests that the FWAQ could be a new tool designed to assess the fear of water. Factor analysis revealed that the scale has three meaningful factors. We found strong reliability for the FWAQ and we believe it to be a robust predictor of the fear of water.

The factor analyses emphasized that the FWAQ has three replicable factors: *Water environment contact*, *Natural force of water*, and *Motion control in water*. This suggests that the fear of water assessment questionnaire interpretations are multifaceted. The first factor, W*ater environment contact*, highlighted the importance of the first steps of learning how to swim, such as exercises of submerging the face and opening the eyes under water. Beside learning and training these two skills, the usage of goggles or mask could be another way to reduce stress and anxiety when in water. Masks or goggles enable unobstructed vision and therefore greater ease in submerging the face (Kapus et al., 2018). Submerging the face might have some positive biomechanical effects in beginner swimmers (e.g., increased buoyancy), which allows them to learn more easily. These aids may help to increase a beginner’s confidence, allowing them to break contact with the bottom of the pool floor or the side of the pool. Moreover, they may help to place swimmers in the proper horizontal body position, thereby simplifying the complex coordination of arms, legs, and breathing (Parker et al., 1999). This could give beginner swimmers additional motivation to try more challenging swimming exercises. However, we should emphasize it may also increase their dependency on those items, which in turn could hinder the acquisition of the two skills and may in fact increase overall fear of the water when a mask or goggles are not available. Therefore, the suggested usage of a mask or goggles should be viewed solely as an aid to decrease initial stress and anxiety from submerging the face and opening the eyes under the water. Beginners should be able to open their eyes under water so that they can better orientate themselves when swimming, in the case of accidently falling into the water without a mask or goggles.

The second factor, *Nature force of water*, gathered the items concerning fear of conditions, which appears in open water. This factor could be expected due to the fact that most drownings occur in open water (Weiss et al., 2010; Tyler et al., 2017). In an open-water situation, water competence can be hindered by cold air, low water temperatures, rough (e.g., waves, surf) water, and clothing (Moran, 2015) – this same effect was found also by Kjendlie et al. (2013), who found an 8% decrease of water competency in a 200-m swim and a 24% decrease in floating performance in 11 year old children. It was also found that life guards swim between 30 to 57% slower when exposed to rough waters (Tipton et al., 2008).

According to this, Stallman et al. (2017) suggested:

1. Water competencies taught have to relate to open as well as closed water environments.
2. Open water competencies can be introduced from an early age through simple tasks, such as water splashing and simulating waves and currents.
3. Pool water safety programs should also simulate rough water.

The third factor, *Motion control in water*, highlighted the importance of the acquisition of two swimming competencies, such us safe entry and gliding (Stallman et al., 2017). The degree of risk upon water entry depends on the task, environment, and the individual (Langendorfer, 2010). Accidental falls into water require the person to hold their breath, reorient themselves, return to the surface, obtain a floating position, rest, and/or start moving in a certain direction. We can safely state that teaching a safe entrance into water (both head and feet first) should be a part of every aquatics and water safety program (Stallman et al., 2017). Due to the meaning of items, which were gathered with this factor (Table 1), the negative correlations between factors were expected. People who were recognized as having a fear of water ranked the items from the first and the second factor with high scores and items from the third factor with low scores.

We suggest swim teachers and coaches consider the use of the FWAQ as means of identification for people with a fear of water; it could also help them find out what frightens an individual or group. This is especially important for personalizing instruction and adapting the learning method when teaching people how to swim (Stillwell, 2011).

## Data Availability Statement

The raw data supporting the conclusions of this article will be made available by the authors, without undue reservation.

## Ethics Statement

The studies involving human participants were reviewed and approved by Faculty of sport, University of Ljubljana. Written informed consent to participate in this study was provided by the participants’ legal guardian/next of kin.

## Author Contributions

TK was in charge of psychological aspects. FM developed the idea and carried out the measurements. SM assisted in statistical processing. JK helped with interpretational issues.

## Conflict of Interest

The authors declare that the research was conducted in the absence of any commercial or financial relationships that could be construed as a potential conflict of interest.

## Appendix

**TABLE A1:** Fear of Water Assessment Questionnaire (FWAQ).

**Item Number**	**Statements**	**Completely disagree**	**Disagree**	**I am not sure**	**Agree**	**Completely agree**
1	When I am in the pool, I am afraid to swim when I am alone	1	2	3	4	5
2	When I am in the pool, I am afraid to swim when I see a lot of people	1	2	3	4	5
3	When I am in the pool, I am afraid to put my face in the water	1	2	3	4	5
4	I cannot swim without goggles	1	2	3	4	5
5	When I am in the pool, I am afraid to see how far the finish edge is	1	2	3	4	5
6	When I am in the pool, I am afraid to open my eyes in water	1	2	3	4	5
7	I am afraid when I lift my legs and float on the surface	1	2	3	4	5
8	I need stairs or shallow water to enter the water	1	2	3	4	5
9	I did not learn how to swim because my home is far away from: Swimming pool, lake, river, or sea	1	2	3	4	5
10	When I see waves, I get scared	1	2	3	4	5
11	When I see open water on the sea, I feel fear	1	2	3	4	5
12	I think I could get lost in the sea during swimming	1	2	3	4	5
13	When I am in a pool, I am afraid when I am not in contact with floor	1	2	3	4	5
14	I am afraid when the water is deep	1	2	3	4	5
15	I could not swim in the river because of water	1	2	3	4	5
16	When my legs sink, I am afraid	1	2	3	4	5
17	I am able to jump legs first into the water from starting block	1	2	3	4	5
18	I am able to jump head first into the water from starting block	1	2	3	4	5
19	I am able to pick up things from the bottom of the shallow pool	1	2	3	4	5
20	I am able to push from the wall and glide on the surface	1	2	3	4	5
